# mHealth or eHealth? Efficacy, Use, and Appreciation of a Web-Based Computer-Tailored Physical Activity Intervention for Dutch Adults: A Randomized Controlled Trial

**DOI:** 10.2196/jmir.6171

**Published:** 2016-11-09

**Authors:** Stefanie Gomez Quiñonez, Michel Jean Louis Walthouwer, Daniela Nadine Schulz, Hein de Vries

**Affiliations:** ^1^ Department of Health Promotion Faculty of Health, Medicine and Life Sciences Maastricht University Maastricht Netherlands; ^2^ CAPHRI-School for Public Health and Primary Care Maastricht University Maastricht Netherlands

**Keywords:** mHealth, eHealth, Web-based intervention, computer-tailored intervention, physical activity

## Abstract

**Background:**

Until a few years ago, Web-based computer-tailored interventions were almost exclusively delivered via computer (eHealth). However, nowadays, interventions delivered via mobile phones (mHealth) are an interesting alternative for health promotion, as they may more easily reach people 24/7.

**Objective:**

The first aim of this study was to compare the efficacy of an mHealth and an eHealth version of a Web-based computer-tailored physical activity intervention with a control group. The second aim was to assess potential differences in use and appreciation between the 2 versions.

**Methods:**

We collected data among 373 Dutch adults at 5 points in time (baseline, after 1 week, after 2 weeks, after 3 weeks, and after 6 months). We recruited participants from a Dutch online research panel and randomly assigned them to 1 of 3 conditions: eHealth (n=138), mHealth (n=108), or control condition (n=127). All participants were asked to complete questionnaires at the 5 points in time. Participants in the eHealth and mHealth group received fully automated tailored feedback messages about their current level of physical activity. Furthermore, they received personal feedback aimed at increasing their amount of physical activity when needed. We used analysis of variance and linear regression analyses to examine differences between the 2 study groups and the control group with regard to efficacy, use, and appreciation.

**Results:**

Participants receiving feedback messages (eHealth and mHealth together) were significantly more physically active after 6 months than participants in the control group (B=8.48, *df*=2, *P*=.03, Cohen d=0.27). We found a small effect size favoring the eHealth condition over the control group (B=6.13, *df*=2, *P*=.09, Cohen d=0.21). The eHealth condition had lower dropout rates (117/138, 84.8%) than the mHealth condition (81/108, 75.0%) and the control group (91/127, 71.7%). Furthermore, in terms of usability and appreciation, the eHealth condition outperformed the mHealth condition with regard to participants receiving (t_182_=3.07, *P*=.002) and reading the feedback messages (t_181_=2.34, *P*=.02), as well as the clarity of the messages (t_181_=1.99, *P*=.049).

**Conclusions:**

We tested 2 Web-based computer-tailored physical activity intervention versions (mHealth and eHealth) against a control condition with regard to efficacy, use, usability, and appreciation. The overall effect was mainly caused by the more effective eHealth intervention. The mHealth app was rated inferior to the eHealth version with regard to usability and appreciation. More research is needed to assess how both methods can complement each other.

**Trial Registration:**

Netherlands Trial Register: NTR4503; http://www.trialregister.nl/trialreg/admin/rctview.asp?TC=4503 (Archived by WebCite at http://www.webcitation.org/6lEi1x40s)

## Introduction

Insufficient physical activity is considered to be a major public health issue worldwide [1,2]. The Dutch public health guidelines recommend adults to engage in moderate- to vigorous-intensity physical activity for at least 30 minutes on at least 5 days per week [3,4]. Studies suggest that sufficient physical activity can effectively prevent numerous chronic diseases and mental health issues [2,4-6]. Lee et al [7] argued that 6% to 10% of worldwide deaths caused by noncommunicable diseases, such as cancer, cardiovascular diseases, and diabetes, can be attributed to physical inactivity. Therefore, there is a need for interventions that increase the level of physical activity and can reach a broad population cost effectively [1].

Empirical research suggests that Web-based computer-tailored interventions are a promising solution [8]. These interventions provide tailored information and feedback via the Internet and therefore have some important advantages. First, Web-based computer-tailored interventions can adapt intervention materials according to the specific situation, characteristics, and needs of an individual and accordingly make information more personally relevant for the individual [9-11]. Second, research has shown that tailored messages are more likely to be read, understood, discussed with others, and remembered by the receiver [12-14]. Third, due to the fact that more and more people are using the Internet to search for health-related information and health advice [15-17], Web-based computer-tailored health interventions offer an effective method to reach a broad population cost effectively [18-22]. Fourth, even though a broad population is targeted simultaneously, each individual can make use of the intervention privately at any given point in time or place [18,23].

Until a few years ago, Web-based computer-tailored interventions were almost exclusively delivered via computer. This medium of delivery has formed the term eHealth (electronic Health). The concept of eHealth has been described as the use of the Internet and related technologies to deliver health-related information and interventions [23]. Even though eHealth has been shown to be an efficient strategy to lower costs and deliver health messages more interactively, it also has several disadvantages. One of the major problems with eHealth interventions is the high percentage of dropout [24,25].

To make interventions even more accessible, and thereby decrease chances of dropout, health promotion professionals are increasingly interested in the use of mHealth (mobile Health). mHealth refers to the delivery of health messages and interventions via mobile phones or tablets by making use of telecommunication and multimedia technologies [26-31]. In the Netherlands, almost 70% of Dutch households use the Internet via mobile phones and approximately 45% use tablets [32]. Based on the increasing usage of mobile phones as a lifestyle device, it has been argued that mHealth might increase the use of interventions and thereby also their efficacy [28,29]. Whereas computers and laptops are relatively stationary, mobile phones and tablets can be carried and used everywhere [33]. People are able to use mHealth independent of time or space, which could improve the usage and evaluation of interventions compared with eHealth [28,31,33].

Most people already use their phones for a variety of personal and work-related matters, such as social networking, calendaring, financial tracking, or emailing [33]. This leads to the assumption that the inclusion of health-related information would be advisable. However, previous research shows some pitfalls of mHealth. First, mobile phone technology is a rapidly changing field that introduces new apps, communication possibilities, and additional gadgets nearly by the day. This makes it difficult for intervention developers to keep up with the newest technologies and interests of their users [34,35]. Second, although using text messaging can be a very effective way of communicating, some intervention messages might be too long or difficult to be presented in such a short manner. This restricted communication can lead to more misunderstandings between the participant and health professional, which in turn can influence the effectiveness of the intervention [36]. And third, both participants and health professionals claim to feel unsure about the safety of private and sensitive information. Although this concern can also arise in the eHealth sector, the inferior but rapidly growing mHealth sector evokes skepticism on both sides [37].

To examine whether mHealth can improve the use and efficacy and reduce dropout rates of Web-based computer-tailored interventions, this study examined the effects of an mHealth and eHealth intervention on physical activity compared with a control group. Both interventions were identical with regard to content but differed in the medium of delivery. The main aim of the study was to examine the efficacy of the 2 versions on physical activity and to compare them with a control group. A secondary aim was to study potential differences in dropout and appreciation of the mHealth and eHealth intervention.

## Methods

### Study Design

The study was a 3-armed randomized controlled trial consisting of a no-treatment control group and 2 experimental conditions (eHealth and mHealth). We recruited participants from a Dutch online research panel and randomly assigned them to 1 of 3 conditions (eHealth, mHealth, or control). Participants were excluded from the study in case of (1) physical conditions hindering engagement in physical activity, (2) pregnancy at the time of recruitment, (3) having a holiday scheduled for more than 5 working days during the study period, and (4) participation in another intervention during the study period.

The baseline measurement took place in April 2014 and the follow-up measurement took place 6 months after baseline (in October 2014). All participants (control, eHealth, and mHealth) were informed about the study by email and asked to complete online questionnaires at 5 points in time: at baseline (T0), 7 days after baseline (T1), 7 days after T1 (T2), 7 days after T2 (T3), and 6 months after baseline (T4: follow-up questionnaire). When a questionnaire had not been completed within 7 days after the invitation email, a reminder was sent. The reminder was sent to prevent dropout and stimulate participants to continue with the intervention. It was not possible for participants to skip sessions, and the next session could only be accessed when the previous one was completed. So when participants received a reminder and accessed the intervention, they continued with the session that followed their last completed session; for example, after session 3, participants could not continue with session 5 until they had completed session 4. Participants received 2 bonus points amounting to €2.50 as an incentive for completing the intervention (the first bonus point after T3, the second one after T4). The 2 intervention groups (eHealth and mHealth) received, additionally to the questionnaires, feedback messages and advice based on their answers to the questionnaires at T0, T1, and T2. Participants allocated to the control condition were also asked to complete all questionnaires but did not receive any feedback or information.

### Power Calculation

To determine the sample size, we conducted a power analysis using G*Power (version 3.1; Heinrich-Heine University Dusseldorf, Germany) [38,39] taking into account an effect size of 0.20, a power of 0.80, and an alpha of 5%. Based on this calculation, a minimum total sample size of 423 (141 participants per condition) was required.

### Intervention

Both the eHealth and the mHealth versions of the intervention were developed using the TailorBuilder software (OverNite Software Europe, Geleen, the Netherlands). Both interventions had exactly the same content. The mHealth intervention was specifically developed for use with a mobile phone, while the eHealth version was developed for use with a computer. Therefore, the intervention within the eHealth condition was delivered via email, whereas in the mHealth condition advice was delivered via short text messages (short message service; SMS). Questionnaires for both groups were sent via email; however, participants allocated to the mHealth group were requested to complete this questionnaire via their mobile phone. Participants in the control condition received an email to inform and remind them that they could assess a questionnaire.

Before starting, participants were clearly instructed that they should use the intervention only via the medium that belonged to their study condition. Participants in the eHealth condition were asked to use the intervention only via the computer and participants in the mHealth version were asked to use the intervention only via their mobile phone or tablet.

We assessed this adherence (use of the intervention) by means of a question in the follow-up questionnaire that asked participants which medium they had used for the intervention. It should be noted that this adherence is correspondingly based on self-reports. It unfortunately was not possible to use the logs of the intervention to assess the medium of use. Hence, we cannot 100% guarantee that the self-reported answers are actually in line with the medium of use. The visual format of the feedback messages was the same in the eHealth and mHealth interventions. In both interventions the feedback messages were merely provided by means of text, without any additional visual content.

The intervention (named *SmartMobiel*) was specifically focused on physical activity as a healthy lifestyle behavior. It was built on an existing eHealth intervention [10] and framed by the I-Change model [40,41] and the health action process approach [42,43]. The main goal of *SmartMobiel* was to stimulate participants’ awareness, ability factors (ie, action plans and goal action), and self-efficacy (see Table 1) to engage in more physical activity. The intervention consisted of 5 successive rounds.

**Table 1:** Theoretical methods, practical strategies, and intervention components of the physical activity intervention *SmartMobiel*.

Determinant	Theoretical method	Practical application	Intervention components
Awareness	Consciousness raising and feedback on performance	Compare baseline physical activity level with physical activity recommendation and current physical activity level	Feedback on participants’ physical activity pattern and sedentary behavior compared with physical activity guideline and additional information on their progress on a weekly basis
Ability factors	Action planning (active learning)	Encourage to formulate action plans	Example of action plan to help formulate appropriate action plans (what, when, where, with whom)
	Preparatory planning (active learning)	Invite to formulate preparatory plans	Suggestion to organize social support (eg, to find a buddy, inform people in the social environment, ask for support, choose a start date)
	Coping planning (active learning)	Encourage to formulate coping plans	Example of coping plan to help formulate appropriate coping plans (if-then)
Self-efficacy	Reinforcement	Compare baseline level in planning, enactment of plans, satisfaction with physical activity, and increased physical activity with current level	Feedback included compliments if planning, etc, were improved; if not successfully improved, feedback included questions stimulating self-reflection

#### Round 1 Feedback: Messages 1-3

The intervention started with a baseline questionnaire (T0) consisting of 38 items concerning demographics, physical activity, sedentary behavior, and psychosocial factors (action planning, intention, satisfaction, and self-efficacy). All measurements were used as input for the tailored feedback messages, which were sent 2 days apart. The main aim of this first round was to inform participants how to successfully plan behavior change regarding physical activity. Based on the baseline questionnaire, participants received 3 feedback messages. The first message provided feedback about participants’ physical activity level. Depending on their reported physical activity level at baseline, the message indicated how their behavior compared with the standards and how they could improve their physical activity level. The second feedback message addressed participants’ intention to engage in physical activity. Finally, the last feedback message of step 1 was focused on planning precisely when, where, and in what type of physical activity participants planned to engage in the following week.

#### Round 2 Feedback: Messages 4-6

Respondents received the second questionnaire (T1) 1 week after baseline, which consisted of questions on physical activity and sedentary behavior (ie, the same questions as in the baseline measurement), intention, and self-efficacy. The main aim of this round was to give participants an overview of their physical activity level and ideas about how to overcome difficulties regarding their behavior change. In this round, 3 tailored feedback messages were sent (message 4, 5, and 6). The fourth feedback message compared participants’ physical activity level with their baseline physical activity level. After 2 days, respondents received the fifth feedback message, which focused on their sedentary behavior and indicated how many hours they sat per week and how they could decrease the time spent sitting. Respondents received a sixth feedback message focusing on self-efficacy with regard to overcoming situations in which it was difficult to be physically active, 5 days after the first follow-up questionnaire had been filled in.

#### Round 3 Feedback: Messages 7-9

During the third round, participants filled in the second follow-up questionnaire (T2). It assessed items regarding physical activity, sedentary behavior, satisfaction, plan enactment, intention, and self-efficacy. The main aim of this round was to encourage participants to act on their plans. Participants received a motivating feedback SMS or email 1 day after the second follow-up questionnaire. After 2 days, respondents received the eighth feedback message, which focused on participants’ habits and goal enactment. Respondents received a last feedback message about their physical activity progress during the intervention, 5 days after the second follow-up questionnaire had been filled in.

#### Round 4 Follow-Up Measurement and Progress Evaluation

The posttest served as a short-term follow-up measurement (T3). This measurement contained 41 items measuring physical activity, sedentary behavior, plan enactment, planning, intention, and self-efficacy. Additionally, we invited both experimental groups to fill in an evaluation questionnaire, consisting of 10 items, which focused on their appreciation of the content of the intervention.

#### Round 5 Final Follow-Up Measurements

This final 6-month follow-up questionnaire contained 35 items and assessed the effects of the intervention on physical activity, sedentary behavior, plan enactment, planning, intention, and self-efficacy.

### Measurements

#### Demographics

At baseline (T0), respondents were asked to indicate their age, sex (1=male; 2=female), marital status (0=no relationship: unmarried without relationship, divorced without new relationship, widowed without new relationship; 1=relationship: married, unmarried in relationship, divorced in new relationship, widowed in new relationship), educational level (1=primary or basic vocational school; 2=secondary vocational school or high school; 3=high vocational school or university), work status (1=student; 2=job: employed, self-employed; 3=no job: unemployed, nonworking, retired), and height (in meters) and weight (in kilograms) to calculate the body mass index (BMI).

#### Outcome Variable

We measured physical activity both at baseline (T0) and at follow-up (T4) with the International Physical Activity Questionnaire (IPAQ) [44-46]. The IPAQ consists of 6 items with a reference period of the past 7 days; participants were asked to indicate how many days per week they had engaged in, respectively, low, moderate, and vigorous physical activity. Additionally, they were asked for how many minutes they usually engaged in these activities on those days. In order to acquire an accurate measure of total physical activity per day, we multiplied the frequency and average duration of vigorous, moderate, and low physical activity and then divided the result by 7.

#### Sociocognitive Variables

We measured all sociocognitive variables (ie, intention, self-efficacy, and action planning) at baseline (T0) and follow-up (T4) using adapted measures from previous studies [47-49] and a 5-point Likert answering scale (1= low to 5= high). Assessment of these variables served as the basis for the feedback messages, as well as correction for potential confounders within the effect analyses. For each variable, we calculated a mean score.

Intention to engage in physical activity was assessed with 4 items (Cronbach alpha=.72). Participants were asked to indicate to what extent they intended to be physically active during the following week; for example, “I intend to be regularly physically active the upcoming week.” The subsequent questions concerned their intention to perform vigorous activities or moderate activities, and finally their intention to walk regularly.

Self-efficacy was measured by means of 6 items (Cronbach alpha=.86). Participants were asked to indicate to what extent they thought they were able to engage in physical activity when encountering difficult situations; for example, “I am going to be physically active next week even though I am stressed.”

Planning was measured by means of 4 items (Cronbach alpha=.89). Plans were related to the participants’ actual planned physical activity; that is, which type of activity, where to be performed, on which days, and for how long. The item stem “I have made a detailed plan regarding...” was followed by the items (1) “which type of physical activity,” (2) “where to exercise,” (3) “on which days to exercise,” and (4) “for how long to exercise.”

Action planning was assessed by 8 items (Cronbach alpha=.85) measuring whether participants planned to execute each of the 8 predefined plans. Action planning included plans that are likely to facilitate physical activity, such as “During the next week, I will buy the necessary equipment to be physically active.”

Plan enactment (T3) was assessed using 8 items (Cronbach alpha=.88) asking participants to indicate the extent to which they actually had executed the 8 actions plans on a 5-point scale. Plan enactment was directly related to the action planning items; for example, “During the last week, I have bought the necessary equipment to be physically active.”

#### Intervention Completion

We measured intervention completion using log file data in order to assess whether participants had completed the separate questionnaires. These scores were summed in order to calculate a total score for intervention use ranging from 0 completed rounds per questionnaire to a maximum of 4 completed rounds per questionnaire.

#### Process Evaluation

At T3, we asked both experimental groups to complete a process evaluation questionnaire. This questionnaire consisted of 10 items that assessed their appreciation of the intervention. One item measured the overall grade of the *SmartMobiel* intervention by asking respondents to give an overall score from 1 (very bad) to 10 (very good). Additionally, we assessed the appreciation of the feedback messages by means of 5 items (1=disagree; 5=agree) to investigate whether the feedback messages were (1) “convincing,” (2) “interesting,” (3) “informative,” (4) “clear,” and (5) “helpful.” Furthermore, we included 1 item using a 5-point scale (1=not appealing at all; 5=very appealing) to measure participants’ appreciation of the intervention design.

### Statistical Analyses

All statistical analyses were performed using IBM SPSS Statistics version 20 (IBM Corporation). We used multiple imputation with 25 iterations to replace missing values on sociocognitive and outcome variables at T0. Additionally, we replaced missing values on BMI and physical activity at T4.

Descriptive statistics and frequencies described the characteristics of the study population. We analyzed differences at baseline using analyses of variance (ANOVAs) with Tukey post hoc tests for continuous variables and chi-square tests with Bonferroni correction for categorical variables.

We analyzed attrition using logistic regression, with attrition at follow-up (T4) as the outcome variable (0=not completed; 1=completed whole intervention), and intervention condition and all baseline variables (ie, age, sex, educational level, BMI, baseline physical activity, and baseline sedentary behavior) as predictors. Process evaluation was analyzed using ANOVA with Tukey post hoc tests to assess the differences between the experimental conditions with regard to usability and appreciation.

Effect analyses were performed using linear regression analyses with the ENTER method. Analyses examined 3 independent effects: (1) intervention (eHealth and mHealth) versus control condition, (2) eHealth versus control condition, and (3) mHealth versus control condition. To analyze the last 2 effects, we recoded the study condition variable into 2 different dummies. We compared each intervention group only with the control group to examine their independent efficacy. All effect analyses were corrected for potential confounders (ie, baseline behavior, baseline differences, and predictors of attrition). We calculated Cohen *d* to assess the size of the possible effects.

### Ethical Approval

The study was approved by the Ethical Committee Psychology of the Faculty of Psychology and Neuroscience at Maastricht University, the Netherlands (ECP-138 08_03_2014) and registered at the Netherlands Trial Register (NTR4503).

## Results

### Sample Characteristics

Table 2 shows the characteristics of the total sample and the baseline differences between the 3 study conditions in terms of demographics, total minutes of physical activity per day, and minutes of moderate to vigorous physical activity. Comparison of baseline variables between groups showed no statistically significant differences.

**Table 2:** Characteristics of the study sample and differences between the study conditions at baseline.

Baseline characteristics		Overall sample (N=373)	eHealth (n=138)	mHealth (n=108)	Control (n=127)	*F* value	*df*	*P* value
Sex (female), n (%)		258 (69.2)	98 (71.0)	77 (71.3)	83 (65.4)	0.74	2	.48
**Educational level, n (%)**						0.36	2	.70
	Low	23 (6.2)	8 (5.8)	4 (3.7)	11 (8.7)			
	Medium	121 (32.4)	48 (34.8)	42 (38.9)	31 (24.4)			
	High	224 (60.1)	81 (58.7)	58 (53.7)	85 (66.9)			
Age in years, mean (SE)		38.69 (11.99)	39.32 (12.10)	38.03 (12.23)	38.55 (11.74)	0.36		.70
Self-efficacy, mean (SD)		3.40 (0.77)	3.35 (0.76)	3.47 (0.78)	3.39 (0.77)	0.73	2	.48
Intention, mean (SD)		3.70 (0.61)	3.72 (0.54)	3.70 (0.65)	3.68 (0.65)	0.10	2	.90
Physical activity level (low, moderate, and high), mean (SD)		54.12 (35.07)	52.72 (36.28)	55.29 (35.20)	54.69 (34.00)	0.18	2	.83

**Table 3:** Attrition analysis.

Baseline characteristics		Odds ratio	*df*	*P* value	95% CI
**Condition (eHealth, mHealth, control)^a^**					
	Condition (eHealth)	2.43	1	.007	1.27–4.62
	Condition (mHealth)	1.29	1	.44	0.68–2.46
Sex (female, male)		2.16	1	.02	1.12–4.14
**Educational level (low, middle, high)^b^**					
	Educational level (low)	1.40	1	.54	0.47–4.14
	Educational level (middle)	1.57	1	.15	0.84–2.92
Age		0.97	1	.009	0.94–0.99
Body mass index		0.98	1	.67	0.91–1.07
Self-efficacy		0.88	1	.53	0.59–1.31
Intention		1.24	1	.40	0.75–2.05
Physical activity (low, moderate, and high)		0.98	1	.18	0.99–1.00
Physical activity (moderate and high)		0.99	1	.049	0.97–1.001

^a^Reference category was the control group.

^b^Reference group was high educational level.

### Attrition Analysis

**Figure 1:**
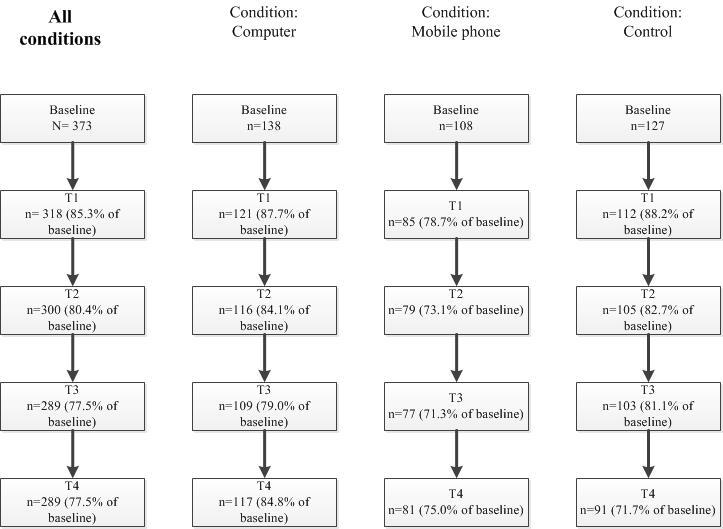
Flowchart of the participation of respondents.

Figure 1 shows the flow of respondents for the overall sample and separately for the 3 study conditions (see Multimedia Appendix 1 [50] for the CONSORT eHealth checklist). Analysis showed that the overall participation rate at follow-up (T4) was 77.5% (289/373). When comparing dropout rates between the 3 conditions, the highest dropout rate was in the control group, in which 71.7% (91/127) of the participants at baseline completed the last follow-up questionnaire. The lowest dropout rate was in the eHealth condition, with a participation rate of 84.8% (117/138).

Attrition analysis (Table 3) showed that respondents were more likely to complete the follow-up assessment when they were in the eHealth condition (compared with the control condition; odds ratio [OR] 2.43, *P*=.007), they were female (OR 2.16, *P*=.02), they were younger (OR 0.97, *P*=.009), and they had lower levels of daily moderate to vigorous physical activity (OR 0.99, *P*=.049). We included the significant predictors of dropout in all further analyses as potential confounders.

### Process Analysis

Results of the process analysis indicate that participants in the eHealth condition evaluated the intervention significantly better than did respondents in the mHealth condition for 3 items: receiving messages, reading messages, and the general clarity of the messages (see Table 4). For the other items, we found no significant differences between the 2 groups.

**Table 4:** Descriptive statistics of the process evaluation.

Variable	Overall sample (n=184) mean (SD)	eHealth (n=109) mean (SD)	mHealth (n=75) mean (SD)	*t* value	*df*	*P* value
Grade given for the whole intervention (range 1–10)	6.35 (1.63)	6.36 (1.60)	6.33 (1.68)	0.10	182	.92
Did you receive the 9 feedback messages?	4.52 (0.84)	4.67 (0.58)	4.29 (1.08)	3.07	182	.002
Did you read the 9 feedback messages you received?	4.60 (0.85)	4.72 (0.59)	4.43 (1.11)	2.34	181	.02
Were the feedback messages believable?	3.43 (0.87)	3.51 (0.83)	3.32 (0.92)	1.46	181	.15
Were the feedback messages interesting?	2.97 (1.05)	2.94 (1.09)	3.01 (0.99)	–0.44	181	.66
Were the feedback messages informative?	3.11 (1.03)	3.02 (1.08)	3.24 (0.96)	–1.43	181	.15
Were the feedback messages clear?	3.90 (0.74)	3.99 (0.74)	3.77 (0.71)	1.99	181	.049
Did the feedback messages help you to be physically active?	2.52 (1.06)	2.48 (1.07)	2.59 (1.05)	–0.66	181	.51
How attractive was the layout of the intervention for you?	3.02 (0.93)	2.99 (0.96)	3.05 (0.88)	–0.45	181	.66

**Table 5:** Intervention effects on the total physical activity at follow-up as assessed by linear regression analyses (multiple imputation).

Intervention^a,b^	B^c^	SE	*df*	*P* value	95% CI
eHealth (1) versus control (0)	6.13	3.61	2	.09	–0.98 to 13.23
mHealth (1) versus control (0)	1.92	4.00	2	.63	–5.95 to 9.79
Intervention (1) versus control (0)	8.48	3.77	2	.03	1.06 to 15.90

^a^In the linear regression analyses the following covariates were included: baseline behavior, sex, age, and baseline moderate and vigorous physical activity.

^b^Outcome variable is average daily physical activity (light, moderate, and vigorous).

^c^B: unstandardized regression coefficient.

### Effect Analysis

Regression analyses showed statistically significant differences between the intervention conditions and the control group for the total amount of physical activity (see Table 5). After 6 months, participants who used the intervention (ie, mHealth and eHealth together) were significantly more physically active than were participants in the control group (intervention groups: mean 56.35 minutes/day; control group: mean 47.79 minutes/day; B=8.48, *df*=2, *P*=.03, Cohen *d*=0.27). We found a small effect that was borderline significant for the difference between the eHealth group and control condition (eHealth: mean 57.91 minutes/day; control group: mean 47.79 minutes/day; B=6.13, *df*=2, *P*=.09, Cohen *d*=0.21) with regard to total physical activity. We found no effect between the mHealth group and the control group (mHealth: mean 54.78 minutes/day; control group: mean 47.79 minutes/day; B=1.92, *df*=2; *P*=.63, Cohen *d*=0.04) with regard to total physical activity. Secondary analyses with complete cases revealed similar results.

## Discussion

### Principal Findings

The aim of this study was to evaluate the efficacy, use, usability, and appreciation of 2 different versions (eHealth vs mHealth) of a Web-based computer-tailored physical activity intervention. Contradicting our hypothesis, the eHealth intervention resulted in better usability and appreciation than did the mHealth intervention. Further, we found no significant differences in use and effects between the mHealth and eHealth versions when compared with a control group. These findings imply that mHealth is not necessarily more suitable than eHealth interventions and even suggest that eHealth should still be preferred.

The effect analyses revealed a significant difference in physical activity when comparing the eHealth and mHealth versions against the control condition. Yet we found no differences in effect between the eHealth and mHealth versions. The effect size for the eHealth version suggests a small effect, but the significance level was only borderline significant due to the small sample size. Recent studies also suggested that the use of mobile phone-based interventions may have positive effects on physical activity and weight loss but did not compare the efficacy of mHealth versus that of eHealth [31,47,48]. In line with our findings, it has been suggested that mHealth may be less suitable to achieve behavior change, since participants in an mHealth condition can use the intervention wherever they are at any given time [49]. One explanation may be that mHealth participants may be more prone to distractions than eHealth users. eHealth users may be more committed to take the time to complete their tasks, whereas mHealth users may have been in distracting surroundings and situations such as supermarkets or public transport, which may lead to skipping or misreading messages. However, this explanation needs more research to demonstrate its applicability. The explanation is in line with the assumption of the elaboration likelihood model of Petty and Cacioppo [51]. The model explains that distraction can result in peripheral route processing rather than in more central processing, which is associated with more (enduring) behavior change [51,52].

The higher dropout rate in the mHealth condition can possibly also be explained by the fact that people are more easily distracted when using their mobile phone. A recent study showed that people tend to use their mobile phones during short waiting times (eg, waiting for the bus, waiting in line at a checkout) [53]. This means, on the one hand, that they use the device frequently; on the other hand, it implies that its use can be short and with many interruptions. Previous studies demonstrated that mobile phone use can distract people from other activities such as driving a car [54,55]. However, ongoing activities and the surroundings might also distract the person from the task he or she is doing on the mobile phone. Distraction might lead to worse performance, as well as to forgetting or neglecting the task completely [54,55]. Furthermore, the possibility of distraction might also explain the finding that the eHealth group evaluated the intervention significantly better than did the mHealth group regarding receiving and reading feedback messages, as well as the clarity of the feedback messages. Elaboration likelihood model research has shown that when information is processed via the peripheral route it is less appreciated by the receiver [56,57]. Furthermore, peripheral route processing can lead to lower motivation to engage with the context of the intervention, which would lead to the lower levels of appreciation [57].

Another explanation could be that, while using a mobile phone is often spontaneous and a direct action that is driven by technology, the use of eHealth might be much more user driven. This means that, whereas participants in the mHealth group might have felt obligated to check their message the moment they received it, regardless of time, place, and concentration, eHealth participants consciously chose to start their computer to check their emails. This feeling of autonomously choosing when to engage in the intervention can lead to more intrinsic motivation and appreciation of the intervention [58]. A different explanation for the low usability and appreciation could be the difference in the technology itself. The intervention was message based, which might have led to more misunderstanding of the feedback messages within the mHealth group than within the eHealth group [37].

### Strengths and Limitations

An important strength is that, to our knowledge, this is the first study that compared an eHealth intervention with an mHealth intervention with regard to efficacy, use, and appreciation.

The first limitation is that all outcome measures were self-reported [59]. Research has shown that self-reported measures, in comparison with objective measures, have both a lower reliability and less validity. However, the IPAQ has been proven to be a reliable and valid measurement of physical activity [46]. Yet replication with other, more objective assessments for measuring physical activity, such as accelerometers, is recommended.

The second limitation is that it was necessary to replace missing values with multiple imputations. Although multiple imputations are often used, there is discussion about how to correctly apply this technique [60]. However, we found the same results regardless of whether we performed the analyses with the multiple imputation or with the completers-only dataset.

The third limitation is that our process analyses were not accompanied by qualitative measurements. For example, by asking participants why they found messages less clear, we could have gained insight into whether the difference between groups was based on technical difficulties only or could be attributed to the other factors.

The fourth limitation is that, because the tested intervention was message based, the results are difficult to generalize to the broader field of mHealth and eHealth.

The fifth limitation is that participants were all recruited from an online panel and were a random sample from the panel. This might make it difficult to generalize the findings from participants who are used to participating in scientific research to the broader population.

Lastly, as pointed out above as well, our sample size was limited. Each condition had approximately 100 participants, and power analyses revealed that we needed at least 141 participants per group to detect standardized effects of 0.20. As the results showed that effect sizes were indeed roughly 0.20, replication of this study with a larger sample is recommended to be able to demonstrate more statistically significant results.

### Conclusion

Based on our results, we can conclude that the eHealth version outperformed the mHealth version of a Web-based computer-tailored physical activity intervention with regard to usability and appreciation, but not with regard to effectiveness.

The eHealth intervention excelled with regard to usability and appreciation compared with the mHealth intervention, and there are indications that the eHealth intervention may have been used more often. However, a study by Morrison et al [61] showed the advantages of combining mHealth and eHealth. They reported that, although their mHealth version did not function as an alternative to eHealth, it enhanced the intervention with regard to perceived accessibility, mobility, and on-the-go gadgets.

We recommend performing more research to assess and develop interventions that combine mHealth and eHealth technologies.

## Appendix

Multimedia Appendix 1 CONSORT-EHEALTH checklist V1.6.1.

## Abbreviations

ANOVA: analysis of variance
BMI: body mass index
IPAQ: International Physical Activity Questionnaire
OR: odds ratio
SMS: short message service
